# Exhaustive screens for disease susceptibility loci incorporating statistical interaction of genotypes: a comparison of likelihood-ratio-based and Akaike and Bayesian information criteria-based methods

**DOI:** 10.1186/1753-6561-1-s1-s25

**Published:** 2007-12-18

**Authors:** Fangyi Gu, Genevieve Monsees, Peter Kraft

**Affiliations:** 1 Program in Molecular and Genetic Epidemiology, Harvard School of Public Health, 677 Huntington Avenue, Building 2, Room 9, Boston, Massachusetts 02115 USA

## Abstract

Several recent papers have suggested that two-locus tests of association that incorporate gene × gene interaction can be more powerful than marginal, single-locus tests across a broad range of multilocus interaction models, even after conservative correction for multiple testing. However, because these two-locus tests are sensitive to marginal associations with either marker, they can be difficult to interpret, and it is not immediately clear how to use them to select a list of the most promising markers worthy of further study. Here we apply single- and two-locus tests to 29 single-nucleotide polymorphisms (SNPs) selected from the dense marker map in the simulated Genetic Analysis Workshop 15 data spanning several candidate regions (the HLA region, the four SNPs flanking "Locus D," and two regions on the q-arm of chromosome 6). We compare the proposed two-locus likelihood ratio tests (LRT) to Akaike and Bayesian Information Criteria (AIC and BIC) for model selection, as well as AIC- and BIC-weighted measures of "SNP importance." The latter provide summary measures of evidence for association between each SNP and disease – including potential interactions with one or more other SNPs – by summing over all one- and two-SNP models. Our results suggest that the LRT using conservative *p*-value criteria were sensitive (but not specific) in identifying associated markers. Standard AIC and BIC criteria were similarly sensitive but not specific. On the other hand, the AIC- and BIC-weighted importance measures yielded a specific but not very sensitive rule for SNP selection. Algorithms incorporating gene × gene interaction to prioritize markers for follow-up will require further development.

## Background

Like other complex diseases, the genetic components of rheumatoid arthritis (RA) are challenging to study. Although the association of RA with specific major histocompatibility complex class II DR loci (HLA-DRB1) alleles on chromosome 6 has been confirmed in numerous populations [1], the exact mechanisms are still unknown. Other susceptibility genes in the same region [2], and other non-HLA genes [3], may play a role. Interactions among loci may contribute broadly to the etiology of RA. For some alleles, the association with RA may only exist on a specific background of other alleles [2]. Therefore, considering multiple loci simultaneously is sometimes necessary for genetic studies of RA, as well as other complex diseases.

Appropriate strategies to discover disease susceptibility loci in the context of genetic association of complex disease involving large numbers of markers are still under development. Marchini et al. [4] and Evans et al. [5] compared standard single-locus tests to two-locus tests of the null hypothesis that disease risk is constant across all combinations of genotypes at two loci (i.e., a test for main effects at either locus or their joint gene × gene interaction). They showed that, across a broad range of two-locus penetrance models, screening all pairwise combinations had comparable or greater power to detect a disease susceptibility locus than single-locus testing.

One drawback to using these two-locus tests is that null single-nucleotide polymorphisms (SNPs) may "piggyback" on a causal SNP with a strong marginal effect because the null hypothesis for the two-locus test assumes neither SNP affects disease risk. Hence, it is not clear how to interpret strongly significant results from two-locus tests, especially in the context of selecting promising SNPs and models to test in an independent data set.

We comprehensively screened 29 SNPs from the simulated dense marker panel on chromosome 6 using both single-marker and two-marker case-control association tests. We considered traditional hypothesis testing (likelihood-ratio tests of nested models) and two model selection/multi-model inference procedures: the Akaike Information Criterion (AIC) and Bayesian Information Criterion (BIC). The latter methods have potential advantages: non-nested models can be easily ranked and measures of "SNP importance" can be constructed by combining information across multiple models containing a SNP. We compare the performance of these approaches by examining their abilities to identify the most promising SNPs among our candidates.

## Methods

All 100 replicates of the Genetic Analysis Workshop (GAW15) simulated case-control data were used, each of which included 1500 cases and 2000 controls. Each case was randomly selected from one of the 1500 affected sib pairs. We considered chromosome 6 both because it contains two simulated causal genes (the DR locus and "locus D" – we were unblinded to the simulated model), and because dense genotyping data were available. Because an exhaustive screen of all pairwise combinations of the 17,820 markers from the dense map on chromosome 6 proved too time-consuming using standard software packages [e.g., SAS PROC LOGISTIC], we selected 29 candidate SNPs: 10 randomly-selected SNPs from the HLA region (which contains the DR locus), 15 SNPs from two candidate regions on 6q, and the four SNPs flanking the locus D (see Table 1).

**Table 1:** Candidate SNP markers

**HLA Region**	*r*^2 ^(DR)^a^	**Flanking Locus D**	**q-arm**	
denseSNP6_3241^b^	0.608	denseSNP6_3915	denseSNP6_11568	denseSNP6_15916
denseSNP6_3426^c^	0.449	denseSNP6_3916	denseSNP6_11611	denseSNP6_11656
denseSNP6_3271	0.404	denseSNP6_3917	denseSNP6_11621	denseSNP6_15886
denseSNP6_3436^c^	0.030	denseSNP6_3918	denseSNP6_15881	denseSNP6_11596
denseSNP6_3281	0.020		denseSNP6_15976	denseSNP6_15961
denseSNP6_3236	0.006		denseSNP6_15921	
denseSNP6_3261^b^	0.003		denseSNP6_16891	
denseSNP6_3251	0.003		denseSNP6_11646	
denseSNP6_3476^c^	0.001		denseSNP6_11666	
denseSNP6_3201^c^	0.001		denseSNP6_11616	

^a^The diallelic *r*^2 ^with DR C allele.

^b^Marginally significant at the study-wide level in over 50% of replicates.

^c^Marginally significant at the genome-wide level in over 50% of the replicates.

Five techniques (likelihood ratio test, AIC, BIC, weighted AIC and weighted BIC) were used to screen these 29 markers for independent or joint association with risk of RA. All SNPs were coded additively (G_i _is the count of minor alleles for marker i, ranging from 0 to 2). We fit single- and two-locus models. For the single marker models, we calculated likelihood ratio tests (LRTs) by comparing the fitted log-likelihood for the null logistic-regression model,

log odds = α,

to the fitted log-likelihood for "Model 1," containing the single marker,

log odds = α + β_i _G_i_.

This leads to a test statistic that has a 1 d.f. chi-square distribution under the null. For the two-marker models, the LRT compares the null model to "Model 2," which includes main effects for each marker and an interaction term:

log odds = α + β_i _G_i _+ β_j _G_j _+ β_ij _G_i _G_j_.

This test has 3 d.f. For every model, the AIC and BIC was calculated as -2 ln(*L*) + 2*p *and -2 ln(*L*) + ln(*n*) *p*, respectively, where *L *is the maximized value of the likelihood function for the estimated model, *p *is the number of parameters in the model, and *n *(3500 for each model) is the number of subjects the model used. Note that in order to appropriately compare models, the LRT, AIC, and BIC all require the same set of subjects be used for all models. In particular, subjects missing any of the tested genotypes should either be excluded from all analyses or missing genotypes should be imputed. This was not an issue for these analyses because there were no missing data in any replicate.

The evidence in favor of a particular model M relative to the null model can be summarized using the change in AIC: ΔAIC_M _= AIC_M_-AIC_0_; ΔBIC_M _is calculated in the same way. By convention ΔAIC_M _values smaller than -10 are taken as strong evidence in favor of model M relative to the null; values between -2 and 2 indicate the two models are roughly equivalent; and values larger than 10 indicate strong evidence against model M relative to the null [6]. These thresholds are motivated by the AIC and BIC weights for each model:

$$AIC\;weight=\frac{\exp(-0.5\Delta AIC_{M})}{\sum_{M}\exp(-0.5\Delta AIC_{M})},$$

where the sum in the denominator is over all models fit. (BIC weights are formed similarly.) These weights can be interpreted as posterior model probabilities under a uniform prior on the discrete set of 436 models considered here [6,7]. (Hence, both ΔAIC_M _and ΔBIC_M _are roughly proportional to the (log) Bayes factor comparing the two models.) The importance of a given SNP can be measured by summing the AIC (BIC) weights for the models containing that SNP.

We fit all possible one- and two-SNP logistic regression models and the null model, resulting in _29_C_2 _+ _29_C_1 _+ 1 = 436 candidate models. For the likelihood ratio test, we consider two significance thresholds. "Genome-wide significance" (*p *< 1.1 × 10^-12^) corresponds to a 0.05 threshold Bonferroni-corrected for all single- and two-locus tests from a genome-wide panel with roughly the same density as the simulated dense chromosome 6 panel (300,000 markers). "Study-wide significance" corresponds to a 0.05 threshold Bonferroni-corrected for the 29 candidate SNPs considered here (*p *< 0.05/435 = 1.1 × 10^-4^).

## Results

**Figure 1:**
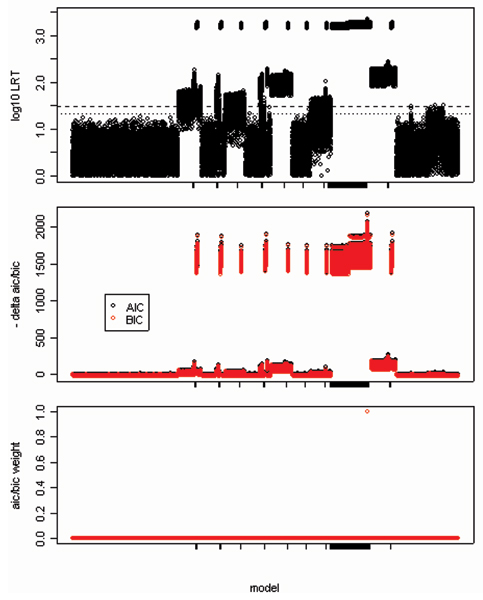
**Log_10 _LRT, ΔAIC/ΔBIC, and AIC/BIC weights**. Log_10 _LRT (top), ΔAIC, ΔBIC (middle), and AIC and BIC weights (bottom) for all 436 models over all 100 replicates, sorted alphabetically (by SNP1 and SNP2) from left to right of the x-axis (from left to right: the null model, the model with denseSNP6_11568 alone, the model with denseSNP6_11568 and denseSNP6_11596,..., the model with SNPs denseSNP6_3437 and denseSNP6_3476). Models containing either s3426 or s3436 are indicated by tick marks on the x-axis. The models containing denseSNP6_3426 and denseSNP6_3436 showed the largest LRT and -ΔAIC (ΔBIC). The dashed line in the top figure is the Bonferroni-corrected genome-wide significance threshold for a chi-squared test with 3 d.f., and the dotted line is the study-wide significance threshold.

Figure 1 shows the LRT, ΔAIC (ΔBIC) and AIC (BIC) weights for each of the 435 non-null models over all 100 replicates. The models containing denseSNP6_3426 and denseSNP6_3436 showed the largest LRT and -ΔAIC (ΔBIC) (top two panels of Figure 1). Because the AIC (BIC) for this model was several hundred units smaller than the next smallest AIC (BIC), the AIC (BIC) weight was ≈1, while the weights for all other models were ≈0 (<10^-10^) (bottom panel of Figure 1).

**Figure 2:**
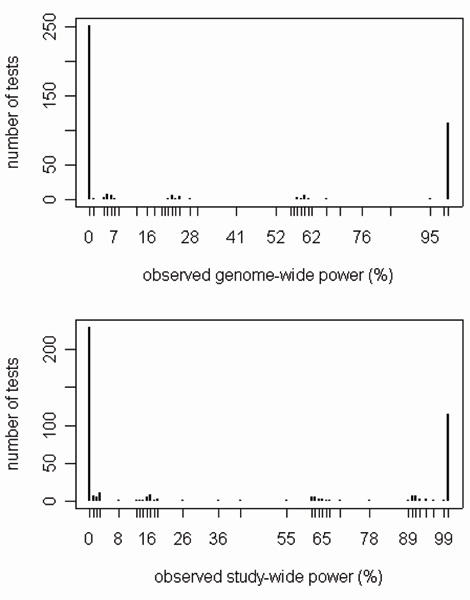
**Observed power for LRT**. Histogram of observed power (percent of replicates significant at genome-wide [top] or study-wide [bottom] level) for all 435 single- and two-locus tests.

One hundred and eleven tests had 100% observed power at the genome-wide significance level, including all 57 tests containing either denseSNP6_3426 or denseSNP6_3436 (Figure 2); 137 tests had greater than 50% observed genome-wide power. The corresponding figures for the study-wide significance level were 114 tests (100% observed power) and 159 tests (>50% observed power).

**Figure 3:**
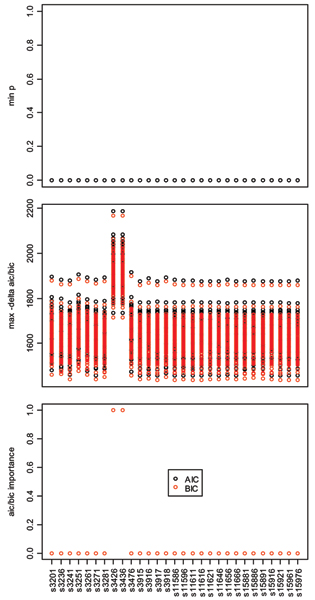
**Minimum LRT *p*-values, maximum -ΔAIC/-ΔBIC values and AIC/BIC importances for each SNP**. Minimum LRT *p*-values (top) and maximum -ΔAIC and -ΔBIC values (middle) over models containing a given SNP. Each point is the value for a given replicate. The bottom panel plots AIC and BIC importances for every SNP over all replicates. Note that for the top and bottom panels, no variation across replicates was observed (e.g., the minimum *p*-value over all tested models was <10^-14 ^for every SNP).

For every SNP, at least one of the models containing that SNP had an LRT *p*-value less than 1.0 × 10^-14^, exceeding the Bonferroni corrected thresholds for both study-wide and genome-wide significance (Figure 3, top panel). Similarly, the values of ΔAIC and ΔBIC were ≤10 for at least one model containing that SNP (Figure 3, middle panel). Thus, tests using standard (and quite conservative) significance thresholds were very non-specific. On the other hand, the AIC and BIC importance measures were quite specific: denseSNP6_3426 and denseSNP6_3436 uniformly had importance scores of ≈1, while all other SNPs had importance scores ≈0 (Figure 3, bottom panel).

## Discussion

Consistent with earlier general arguments [4,5], we found that many two-locus tests for genetic association had perfect power to detect associations between RA and SNPs near the DR locus in the GAW15 simulated data even after very conservative genome-wide Bonferoni correction for multiple comparisons. Further, the test that considered SNPs denseSNP6_3426 and denseSNP6_3436 together provided greater evidence for association (smaller *p*-values, smaller AIC and BIC values) than the tests considering either SNP alone. However, while powerful, these two-locus tests were not very specific: every candidate SNP – even those on the q arm of chromosome 6, which were not in linkage disequilibrium with any of the simulated causal variants – was significantly associated with risk of RA at the genome-wide level when tested jointly with either denseSNP6_3426 or denseSNP6_3436. This is because the joint test can have good power even when only one of the two SNPs affects disease risk. Similarly, the change in AIC or BIC relative to the null model was not very specific: every candidate SNP was included in at least one model that had an AIC and BIC far smaller than that of the null model. LRTs and AIC/BIC values may be useful for ranking markers relative to each other (c.f. the middle panel of Figure 3), but they may provide little guidance on the absolute level of evidence for association (e.g., how many SNPs are worth further investigation).

On the other hand, the AIC- and BIC-weighted importance measures were quite specific but not particularly sensitive – only SNPs denseSNP6_3426 and denseSNP6_3436 had non-negligible importance values, although five of the ten HLA-region SNPs yielded significance near the marginal genome-wide level and six showed marginal study-wide significance in greater than 50% of the replicates (Table 1). This is because the AIC and BIC model weights are calculated relative to the other models considered; in this case, the evidence for model denseSNP6_3426 and denseSNP6_3436 relative to every other model was so strong its weight was ≈1. This suggests an iterative process might be helpful in selecting SNPs for further study: select those SNPs with importance above a given threshold; remove them from the data set and recalculate SNP importance values (note that this does not require any likelihoods to be recalculated); select any SNPs whose importance exceeds the recalculated threshold and repeat the selection process until no remaining SNPs exceed the recalculated threshold.

Appropriate statistical strategies are currently being sought for the consideration of statistical interactions that may increase power, and are sometimes necessary, to detect a causal variant. However, more work is needed to develop sensitive and specific procedures for selecting promising markers from a large-scale association study, considering the great cost of genotyping large sample sizes typical of second stage genome-wide association scans. In the GAW15 simulated data (candidate SNPs from the dense marker map on chromosome 6), we found that naively applying conservative Bonferoni-corrected *p*-value thresholds to the model-free two-locus test statistic proposed by Marchini et al. yielded sensitive but not specific tests of association. On the other hand, our proposed straightforward application of AIC or BIC variable importance measures yielded a specific but not very sensitive rule for SNP selection. These results are driven in large part by the genetic architecture of the simulated RA trait. The signal at the DR locus is strong enough and the sample size large enough to produce highly statistically significant results (*p *< 1 × 10^-14^) for loci in the HLA region even when a null marker and its interactions are included in the model; similarly, small differences in the linkage disequilibrium structure between HLA markers and the DR locus are amplified here so that a single pair of HLA markers provided a far better fit than any other single marker or pair of markers. The performance of these methods in other situations – notably when the sample size is smaller or the signal for association is much more modest, as is expected for most complex disease loci – requires further investigation.

In real situations, missing data can lead not only to a loss of power, but may also make it impossible to compare models with different SNPs, as we have described in our Methods section. In these circumstances, we recommend imputing missing SNPs using the local haplotype structure to create a complete data set prior to analysis, to account for missing data problem while minimizing the loss of information.

## Competing interests

The author(s) declare that they have no competing interests.
